# Non-linear correlation analysis between internet searches and epidemic trends

**DOI:** 10.3389/fpubh.2025.1435513

**Published:** 2025-04-04

**Authors:** Yongzhang He, Lingshi Ran, Yang Wang, Fengxiang Huang, Yixue Xia

**Affiliations:** Research Center of Network Public Opinion Governance, China People's Police University, Langfang, China

**Keywords:** COVID-19, internet, non-linear dynamics, epidemiological forecasting, evolutionary patterns

## Abstract

**Introduction:**

This study uses a non-linear model to explore the impact mechanism of change rates between internet search behavior and confirmed COVID-19 cases. The research background focuses on epidemic monitoring, leveraging internet search data as a real-time tool to capture public interest and predict epidemic development. The goal is to establish a widely applicable mathematical framework through the analysis of long-term disease data.

**Methods:**

Data were sourced from the Baidu Index for COVID-19-related search behavior and confirmed COVID-19 case data from the National Health Commission of China. A logistic-based non-linear differential equation model was employed to analyze the mutual influence mechanism between confirmed case numbers and the rate of change in search behavior. Structural and operator relationships between variables were determined through segmented data fitting and regression analysis.

**Results:**

The results indicated a significant non-linear correlation between search behavior and confirmed COVID-19 cases. The non-linear differential equation model constructed in this study successfully passed both structural and correlation tests, with dynamic data fitting showing a high degree of consistency. The study further quantified the mutual influence between search behavior and confirmed cases, revealing a strong feedback loop between the two: changes in search behavior significantly drove the growth of confirmed cases, while the increase in confirmed cases also stimulated the public's search behavior. This finding suggests that search behavior not only reflects the development trend of the epidemic but can also serve as an effective indicator for predicting the evolution of the pandemic.

**Discussion:**

This study enriches the understanding of epidemic transmission mechanisms by quantifying the dynamic interaction between public search behavior and epidemic spread. Compared to simple prediction models, this study focuses more on stable common mechanisms and structural analysis, laying a foundation for future research on public health events.

## 1 Introduction

Since the outbreak of the COVID-19 pandemic in late 2019, it quickly became a global focus (1). This global epidemic resulted in significant human and economic losses worldwide (2). During the outbreak of global infectious diseases like the COVID-19 pandemic, traditional bottom-up data collection methods face significant challenges: delays in data collection and processing make it difficult to keep up with the rapid development of events, thereby affecting decision-makers' ability to understand and respond to situations promptly. In response to such global infectious diseases, scholars have proposed various monitoring methods to intervene quickly and accurately, reducing the harm caused by crisis outbreaks (3).

This study focuses on the application of internet search data. The immediacy and dynamic nature of internet search data in today's era offer a new perspective for capturing the focus and behavioral changes of the public. These data cannot only reflect the public's information needs and points of interest in real-time but also reveal people's expectations and concerns about impending events.

However, existing research mainly focuses on analyzing the relationships between variables based on specific data and model methods. These methods typically focus on fitting model parameters using specific data and variable dimensions and adjusting accordingly. Therefore, when data or dimensions change, the parameters of the model change as well. This means that these models largely rely on the specific characteristics of the data and cannot truly uncover the underlying logic between variables.

In contrast, this study emphasizes exploring the impact mechanism of variables on themselves and the rate of change between variables. By establishing a derivative-based non-linear model, we aim to reveal the rate of interaction between search behavior and the number of confirmed cases and to uncover a stable influence mechanism, rather than merely identifying simple correlations between data. This underlying logical structure has greater generalizability and can be applied across different datasets and scenarios, with the structure and operators of the equations remaining unchanged regardless of how the parameters vary. Thus, the contribution of this paper lies in providing a general framework that can be adapted to different infectious diseases and datasets, exploring the common mechanisms in epidemic spread.

### 1.1 The impact of the COVID-19 pandemic on human behavior

During the COVID-19 pandemic, the restrictions on people's mobility led to a sharp increase in the use of social networks. The outbreak and spread of the pandemic also intensified internet users' demand for information related to COVID-19. Additionally, because of the pandemic's long duration and profound impact, the search data generated during this period was particularly rich. Wang et al. used the BERT model to analyze the negative sentiment related to COVID-19 on Chinese social media, revealing changes in public emotions and psychological responses during the pandemic (4). Bagarić et al. developed a simulated search engine, “Foogle,” to explore how the presentation of information and individuals' fear of the pandemic influenced search behavior (5). Their research found that users were more inclined to click on information related to the severity of the pandemic during a major health crisis. In the early stages of vaccine rollout, An et al. studied the search behavior of the American public regarding COVID-19 vaccines, finding that search demand evolved as vaccine-related information was updated (6). A systematic review by Masaeli et al. showed a significant increase in internet addiction during the pandemic, closely related to long-term social isolation and health anxiety (7).

These studies not only illustrate the profound impact of the COVID-19 pandemic on individuals' emotions, information needs, and behavior but also highlight the significant changes in the way public health information is disseminated and how people respond to it. In the context of a global crisis, the surge in online searches provides an entry point for further analysis of how internet search data can be utilized for monitoring the spread of infectious diseases.

### 1.2 The relationship between search data and infectious diseases

The rapid development of internet technology has led to the widespread use of search data as an emerging data source in the monitoring and prediction of infectious diseases. Early studies predominantly employed simple linear regression models to explore the relationship between search frequency and disease outbreaks. Ginsberg et al. (8) were pioneers in this field. By analyzing Google search query data, they found a strong correlation between search frequency and the number of flu cases during outbreaks. Although their model used a simple linear approach, which could not capture the complex non-linear dynamics of flu transmission, their pioneering work provided a new perspective for researchers in infectious disease studies.

With the continuous advancement of research, scholars began to recognize the limitations of simple linear models and transitioned to methods such as time series analysis and statistical models. For example, Zhou et al. employed a linear time series model that integrated Baidu search engine data and news reports to monitor infectious diseases in China (9). Bhattacharya analyzed the time correlation between Google Trends data and disease data, using forecasting methods to predict disease outbreaks in real-time (10).

Subsequent studies acknowledged that more complex nonlinear models were better suited to capturing the dynamic nature of disease transmission. For example, Alsmadi and Obeidat used a Support Vector Machine (SVM) model, combining Google search data with hepatitis case reports, significantly improving prediction accuracy (11). Wisnieski et al. applied a rolling window negative binomial regression model to analyze the time-lagged relationship between Google Trends and Lyme disease cases in the U.S., successfully capturing dynamic changes between search data and disease outbreaks across different time periods (12). This demonstrated the effectiveness of non-linear models in disease prediction.

During the COVID-19 pandemic, the application of non-linear methods became even more widespread and sophisticated. Abbas et al. used dynamic correlation analysis to examine the relationship between COVID-19-related symptoms and confirmed cases and deaths (13). This study demonstrated how Functional Principal Component Analysis (FPCA) could capture the spatiotemporal evolution of COVID-19 transmission. Galido et al. analyzed the relationship between search trends for protective measures such as masks and handwashing and case numbers during the pandemic (14). Using Google Trends data and Spearman correlation analysis, the study revealed a direct relationship between preventive measures and pandemic transmission, highlighting the application of non-linear models in public health surveillance. Toomre et al. further explored the long-term association between Google search data and COVID-19 hospitalization and death rates by calculating cross-correlation, finding that search volumes for “loss of smell” and “loss of taste” peaked 2 to 3 weeks before surges in case numbers (15).

In both traditional infectious disease research and studies related to COVID-19, researchers have progressively improved their methods from simple linear models to more complex non-linear models, successfully capturing non-linear trends between search data and case numbers. These advancements have allowed for a better understanding of the complex dynamics in disease transmission. However, most studies remain at a descriptive level and have not fully quantified the interactions between different variables, nor have they explored how these variables interact, the strength of these interactions, or how they influence the evolution of the epidemic. Although some studies [e.g., Wisnieski et al. (12)] introduced temporal dimensions, these analyses were often focused on shorter time periods, which may not fully reflect the complexity and variations across the entire course of the epidemic. Additionally, prior research often employed detailed classifications of population groups, which, while informative, could limit the generalizability and interpretability of the models.

Based on these observations, this study proposes the following innovations:

**(1) Research on the mechanism of variable influence:** This paper constructs a derivative-based non-linear model to quantify influence functions, delving into how variables affect their own rate of change, rather than merely focusing on parameter fitting or analyzing surface relationships between variables. The study aims to uncover a stable self-influence mechanism and build a generalizable equation structure.
**(2) Temporal evolution analysis**: This study uses data from a complete cycle (3 years) and divides time into different stages to analyze the evolving relationship between search behavior and confirmed cases. This method allows for a more detailed understanding of how the correlation evolves over time, providing a clearer sense of the long-term interaction between search behavior and confirmed cases.
**(3) Simplification of population classification**: This study abandons overly detailed classification of different population groups, instead merging various categories to create a more practical and applicable model. This enhances the model's adaptability to real-world scenarios, making the research results more directly applicable to public health strategies and policies.

## 2 Data pre-processing

This study collected data from two platforms: the Baidu Index, used to crawl the COVID-19 search index, and the official website of the National Health Commission of the People's Republic of China, from which the confirmed case data was crawled.

The Baidu Index is a data platform based on the behavior of netizens. It recorded a large volume of “COVID-19” related search information during the pandemic, reflecting public concern. As the search engine platform with the highest market share in China, Baidu leads both the PC and mobile sectors in terms of user penetration, with a mobile penetration rate as high as 88.4% according to the CTR 2023 China Search Engine Industry Research Report (16). Therefore, Baidu Index data holds high representativeness and authority, accurately reflecting the search behavior of Chinese internet users. We crawled the daily search frequency for the keyword “COVID-19” in the Baidu Index from February 29, 2020, to December 23, 2022, referring to it as the COVID-19 search index (SI). Although search data may be influenced by personalized recommendation algorithms, Baidu's extensive coverage ensures that its data remains a key indicator for measuring public interest.

**Figure 1:**
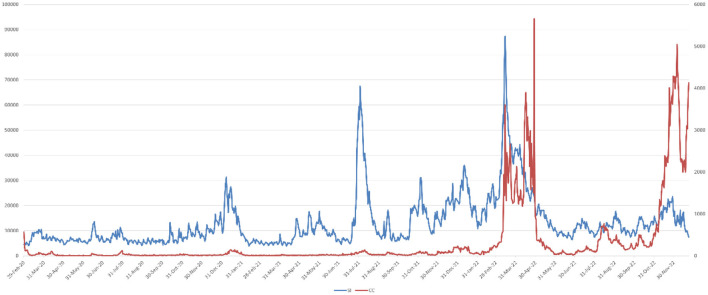
Line chart of SI and CC data from 2020/2/29–2022/12/23.

The National Health Commission of the People's Republic of China is the most authoritative health management institution in China, responsible for formulating and implementing public health policies and for collecting and publishing epidemic data nationwide. During the COVID-19 pandemic, the official website of the National Health Commission became the primary channel for the public to obtain the latest and most authoritative epidemic information. Its data collection mechanism is rigorous, involving direct case reporting from municipal medical institutions and testing centers across the country, followed by multiple layers of verification and consolidation before publication, ensuring data timeliness and accuracy. Although any data collection system may involve a degree of error or delay, the National Health Commission's data remains the most reliable source of official epidemic reporting. We crawled the daily number of confirmed COVID-19 cases (CC) from the official website from February 29, 2020, to December 23, 2022. This dataset covers nationwide case information and is both comprehensive and widely representative. Figure 1 shows the crawled data.

Through the observation of Figure 1, it can be noticed that the data with global periodicity exhibits a certain level of correlation, with their peak fluctuations roughly similar.

**Figure 2:**
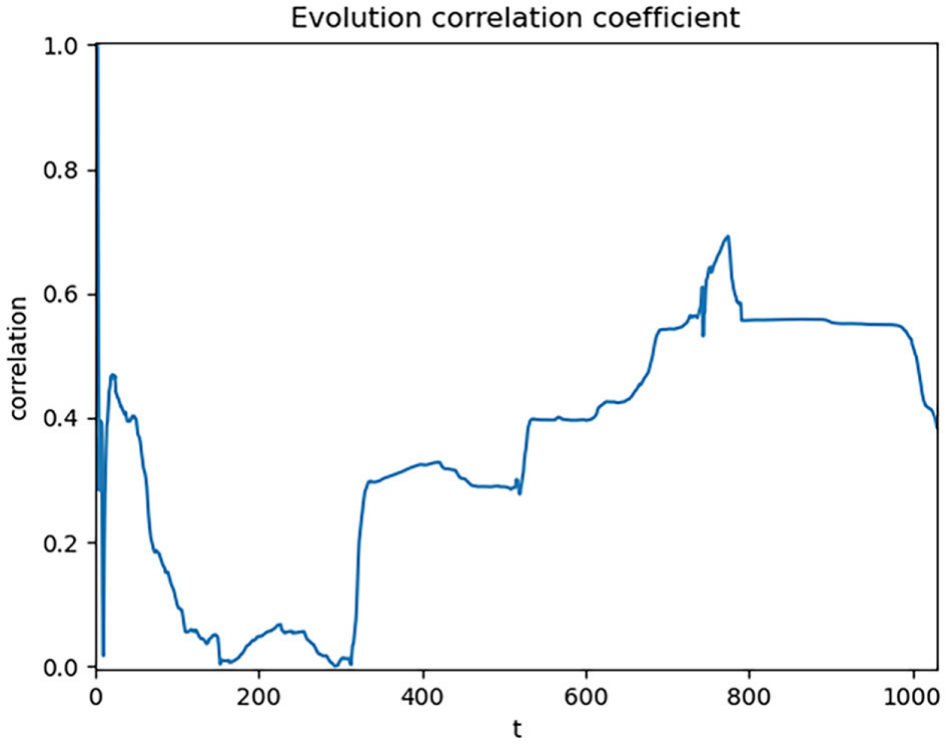
Dynamic correlation coefficient of global data.

In order to further validate the research value of the selected data, a correlation test is needed. We choose the Pearson correlation coefficient to evaluate the correlation between the two variables, as it provides the magnitude of direct correlation between variables, facilitating the assessment of their linear relationship. Because this method requires at least three basic data points for correlation analysis, we start from the third day of SI and CC data, adding 1 day of data each time, and calculating the Pearson correlation coefficient C. The results are shown in Figure 2.

**Figure 3:**
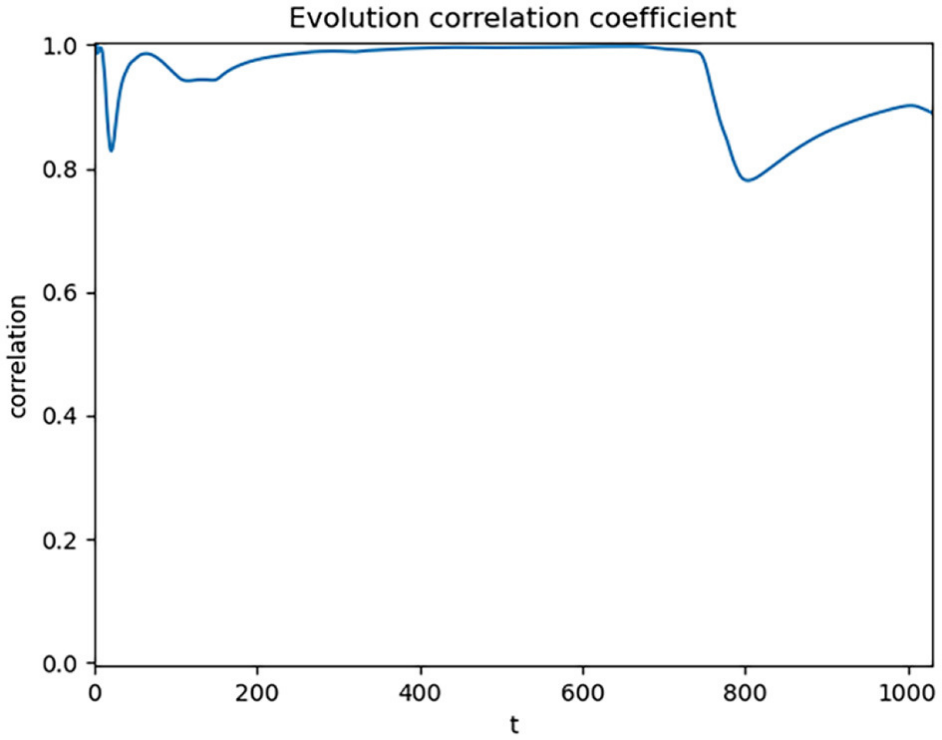
Dynamic correlation coefficient of global cumulative data.

When observing the data, we can see that the correlation coefficient fluctuates around 0 and does not exhibit a clear correlation. The final result shows a correlation coefficient of only 0.38 between the two datasets, which does not meet the requirements of this study. Upon investigation, we find that the problem may stem from the methodological limitations of using single-day data for correlation analysis. This study examines the influence between the SI and CC, and single-day data may not fully reflect this long-term cumulative effect. Therefore, we adopted a strategy of using cumulative data. The use of cumulative data is because we are concerned with the “evolution process” of the two types of data rather than the “change process.” Evolution includes history, which better reflects the “temporality” of the data, while change focuses more on the “spatiality” of the data and does not include history. Therefore, to consider historical influences in the data, we accumulated the data. By accumulating the data, the trend of data evolving over time can be better reflected. The correlation results of the cumulative data are shown in Figure 3.

After the cumulative processing, we observed a significant enhancement in the correlation between the SI and CC, with a correlation coefficient as high as 0.89. However, we observed a decline in the correlation around day 800, dropping to approximately 0.8. By analyzing the data in Figure 1, along with reports from the National Health Commission and related information, we identified that this decline might be related to the emergence of the Omicron variant. Starting on March 12, 2022, the number of asymptomatic cases in Shanghai surged, leading to a city-wide lockdown implemented on March 28. During the peak of confirmed cases from March 12 to April 29, Shanghai accounted for about 90% of the national daily confirmed cases. This abnormal data fluctuation caused a drop in the overall correlation.

**Figure 4:**
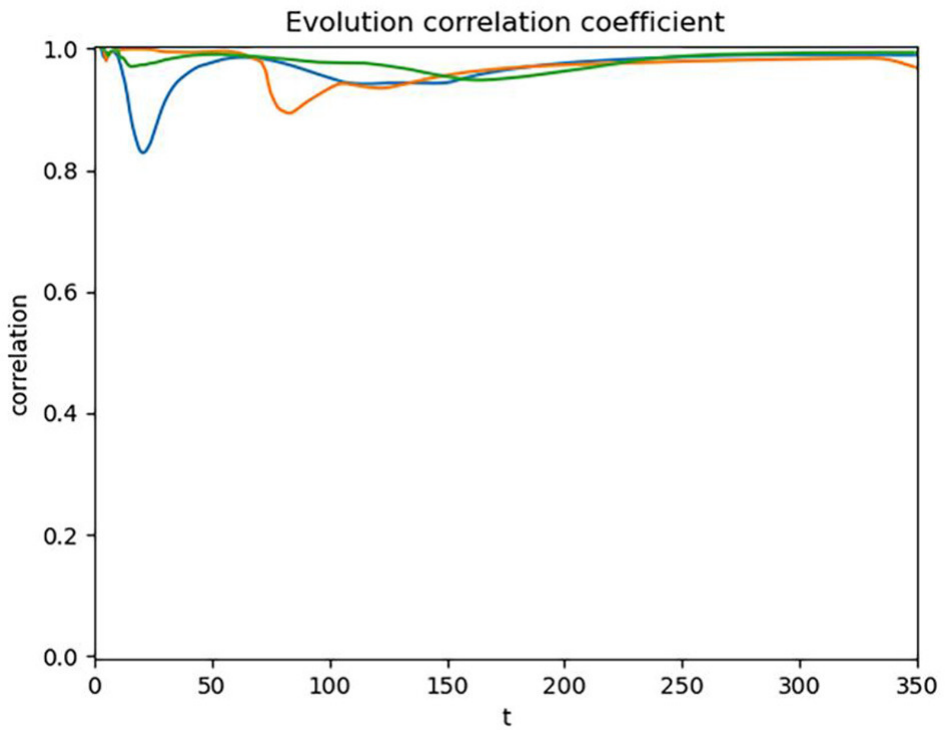
Dynamic correlation coefficient of segmented cumulative data.

Given the occurrence of such abnormal fluctuations in the data and their impact on the overall correlation analysis, we deemed it necessary to segment the data further to eliminate the interference of localized fluctuations on the overall analysis. Specifically, we segmented the data by year into three segments: the first segment from February 29, 2020, to December 31, 2020; the second segment from January 1, 2021, to December 31, 2021; and the third segment from January 1, 2022, to December 23, 2022. Similarly, to validate the rationale of this operation, we conducted a correlation test on the segmented data. The results of the correlation test are shown in Figure 4.

The result shows that after segmenting the data, the correlation coefficient further increased to 0.98, indicating a better correlation in the segmented data. This finding not only validates the effectiveness of our segmentation method but also reveals the extremely close dynamic relationship between public search behavior and the actual development of the pandemic in different phases.

Through the preprocessing of the data, we have shifted our research focus from the daily data of SI and CC throughout the full cycle to the cumulative data segmented by periods. This helps us delve deeper into the patterns between the data, laying the groundwork for building models in the next steps.

## 3 Building the basic model

The development of the COVID-19 pandemic exhibits distinct cyclical characteristics, which can be clearly divided into the “latent period,” the “outbreak period,” and the “decline period” (17). This process is clearly reflected in the changes in confirmed cases data. Correspondingly, people's COVID-19-related internet search behavior synchronizes with the cyclical nature of the pandemic development and also demonstrates similar lifecycle characteristics.

In this study, we temporarily consider only the impact of search data on confirmed cases, without accounting for other factors such as regional differences or emotional responses. Building upon this, our study selects the logistic growth model framework to construct an initial model to analyze the underlying relationships and patterns. This model framework allows us to explore the intrinsic relationships between COVID-19 search behavior and the pandemic's lifecycle stages, providing valuable insights into their dynamics.

### 3.1 Constructing the basic model

Since the behavior of SI and CC can be approximated as smooth and continuous over time, it is assumed that their cumulative values are both continuous and differentiable functions of time t, denoted as *x*_*i*1_(*t*) and *x*_*i*2_(*t*) respectively. Furthermore, an upper limit variable is added as a constraint, which refers to the maximum limit that the variable can grow to under environmental or other constraints, with the maximum SI and CC limits denoted as *K*_*i*1_ and *K*_*i*2_, thus the remaining spaces are $\left(1-\frac{x_{i1}}{K_{i1}}\right)$ and $\left(1-\frac{x_{i2}}{K_{i2}}\right)$ respectively. Following the logistic modeling approach, we can establish the foundational models for both as:


(1) $$\{\begin{array}{c} \frac{dx_{i1}}{dt}=r_{i1}x_{i1}\left(1-\frac{x_{i1}}{K_{i1}}\right)\\ \frac{dx_{i2}}{dt}=r_{i2}x_{i2}\left(1-\frac{x_{i2}}{K_{i2}}\right) \end{array}$$

where *i* = 1, 2, 3, corresponding to three data segments, with *r* representing the natural growth rate, which refers to the rate at which the variable grows over time in the absence of other limiting factors we assume that *r*0 and is constant. $\frac{dx_{i1}}{dt}$ and $\frac{dx_{i2}}{dt}$ represent the rate of change of *x*_1_ and *x*_2_with respect to time *t*, respectively. *r*_*i*1_ and *r*_*i*2_ are the natural growth rates for the SI and CC, with initial values *x*_*i*1_(0) and *x*_*i*2_(0) respectively. In this model, we temporarily disregard the potential interplay between these two variables, initially assuming their growth rates are directly proportional to the current quantity and available growth space.

### 3.2 Validating the basic model

Having obtained the basic model, the next step is to further validate the rationality and accuracy of the model. The method for estimating model parameters is based on the differential regression approach described in the paper by Lan et al. (18). We will transform the model into corresponding difference equations:


(2) $$\{\begin{array}{c} \Delta x_{i1}\left(n\right)=r_{i1}x_{i1}\left(1-\frac{x_{i1}}{K_{i1}}\right)=r_{i1}x_{i1}-\frac{r_{i1}}{K_{i1}}x_{i1}^{2}\\ \Delta x_{i2}\left(n\right)=r_{i2}x_{i2}\left(1-\frac{x_{i2}}{K_{i2}}\right)=r_{i2}x_{i2}-\frac{r_{i2}}{K_{i2}}x_{i2}^{2} \end{array}$$

Where Δ*x*_*i*1_(*n*) = *x*_*i*1_(*n*)−*x*_*i*1_(*n*−1) *and Δx*_*i*2_(*n*) = *x*_*i*2_(*n*)−*x*_*i*2_(*n*−1), meaning that Δ*x*_*i*1_(*n*) and Δ*x*_*i*2_(*n*) correspond to the single-day data of SI and CC on the n-th day, respectively. By transforming the differential equations into a set of difference equations, we can convert the parameter fitting problem of the differential equations into solving the regression coefficients of the difference equations. It was observed from the left-hand side of Equation 2 that the difference Δ*x*_*i*1_(*n*) exhibited a bivariate linear relationship with *x*_*i*1_ and $x_{i1}^{2}$, while Δ*x*_*i*2_(*n*) exhibited a bivariate linear relationship with *x*_*i*2_ and $x_{i2}^{2}$. Applying bivariate linear regression analysis allows us to obtain the regression coefficients *r*_*i*1_,$-\frac{r_{i1}}{K_{i1}}$,*r*_*i*2_, and $-\frac{r_{i2}}{K_{i2}}$, thus obtaining the model parameters *r*_*i*1_, *r*_*i*2_, *K*_*i*1_, and *K*_*i*2_.

After obtaining the coefficients, we need to conduct model verification. In this study, global static regression analysis was conducted on three sets of data, with each set comprising two equations, resulting in a total of six regression analyses. To ensure the reliability of the regression results, three test standards were set as the basis for the test results:

(1) Structural test: the natural growth rate *r*_*ij*_>0, the upper limit *K*_*ij*_>0, and $-\;\frac{r_{ij}}{K_{ij}}$ < 0;
(2) Correlation test: the fitting index R-squared >0.25 for the regression analysis is considered to pass the data correlation test.
(3) Significance test: if the significance test index *P-*value in the regression analysis is < 0.05, it indicates that the regression coefficients have strong significance, considered to pass the significance test.

The results of the regression analysis are shown in the Table 1.

**Table 1:** Regression analysis results of six sets of modeling data.

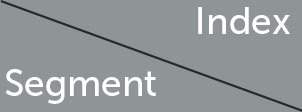		** $-\frac{r_{im}}{K_{im}}$ **	**$-\frac{r_{im}}{K_{im}}$ *P*-value**	***R* ^2^**
First segment	*x*_11_	0.009797	3.56E-44	−2.6E-09	1.78E-12	0.813048
	*x*_12_	0.012924	2.48E-09	−1.6E-06	1.05E-05	0.209199
Second segment	*x*_21_	0.010161	1.02E-31	−1.5E-09	3.06E-11	0.623541
	*x*_22_	0.004099	2.5E-06	2.25E-07	0.006953	0.616017
Third segment	*x*_31_	0.014691	6.92E-50	−2.2E-09	2.74E-35	0.620284
	*x*_32_	−0.00058	0.639258	4.4E-08	1.56E-10	0.506886

Observing the results, among the 6 sets of data, the data for *x*_*i*1_ passed all three tests. However, the data for *x*_*i*2_ did not pass all three tests. Specifically, in the *x*_12_ group, *R*^2^ = 0.209 < 0.25, indicating failure in the correlation test; in the *x*_22_ group, the coefficient of $\frac{r_{22}}{K_{22}}$ is positive, failing to meet the structural test requirement of being < 0; in the *x*_32_ group, the coefficients *r*_32_ and $\frac{r_{32}}{K_{32}}$ are of opposite signs, also failing to meet the structural test requirement, and the corresponding *P*-value for *r*_32_ is 0.64, which does not meet the specified significance test requirement of being < 0.05.

When investigating the reasons for the data not meeting the predefined test standards, we realized that the uncertainty in static data fitting is a key factor. Although static analysis provides us with preliminary understanding, it overlooks the dynamic nature of data over time. Therefore, to more accurately verify the effectiveness of the model, this study adopted a dynamic fitting method, which involves gradually adding data points for model validation to simulate the cumulative effect of data over time. Specifically, starting from the third data point of each segment, one data point was added for model validation each time. To test whether the structure meets the research requirements, we set the Conformity Rate (CR), which records the number of data points that pass the three tests through dynamic regression analysis. A higher CR indicates a better fitting effect of the model.


(3) $$CR=\frac{N_{p}}{N_{total}}$$

Where *N*_*p*_ represents the number of passes in the test, and *N*_*total*_ represents the total number of regression analyses for that structure. The test results are shown in the Table 2.

**Table 2:** Dynamic regression analysis results.

**Segment**		**CR**
First segment	*x*_11_	98%
	*x*_12_	41%
Second segment	*x*_21_	89%
	*x*_22_	77%
Third segment	*x*_31_	95%
	*x*_32_	82%

The results of dynamic fitting show that the majority of structures exhibit extremely high conformity rates, particularly in the first and third segments of data, with CR values reaching 98% and 95% respectively. As for the *x*_12_ group, which had a fitting effectiveness of 41%, analysis revealed that most failures were due to the correlation test not passing, with *R*^2^ not reaching the predefined 0.25, although most were above 0.2. We separately conducted structural and significance tests for these cases, and the results showed a high conformity rate of 96%. This indicates that although only half of the six static tests for segmented data passed, the overall dynamic fitting effect is very good. All regression analyses passed structural tests, indicating that the assumptions about the model structure have been fully validated.

## 4 Non-linear correlation model

### 4.1 Common equations

After validating the rationality of the basic model, we continue to delve into the correlation between the two types of data. The influence of the cumulative CC on the growth rate of SI is quantified as function *f*_1_, while the influence of the cumulative SI on the growth rate of the CC is quantified as function *f*_2_. Therefore, *f*_1_ and *f*_2_ can be considered as functions solely containing *x*_*i*1_ and *x*_*i*2_. Based on this assumption, we obtain further model expressions:


(4) $$\{\begin{array}{c} \frac{dx_{i1}}{dt}=r_{i1}x_{i1}\left(1-\frac{x_{i1}}{K_{i1}}\right)+f_{1}\left(x_{i1},x_{i2}\right)\\ \frac{dx_{i2}}{dt}=r_{i2}x_{i2}\left(1-\frac{x_{i2}}{K_{i2}}\right)+f_{2}\left(x_{i1},x_{i2}\right) \end{array}$$

The first half of the model represents “self-growth,” while the latter part represents “mutual interaction.” The functions *f*_1_(*x*_*i*1_, *x*_*i*2_) and *f*_2_(*x*_*i*1_, *x*_*i*2_) represent the impact functions. So, what do these impact functions look like?

#### 4.1.1 Analysis of impact function structure

Firstly, we need to determine the degree of the impact functions. Based on the basic model from Chapter 3 $\frac{dx}{dt}=rx\left(1-\frac{x}{K}\right)=rx-\frac{r}{K}x^{2}$, we know that the rate of change $\frac{dx}{dt}$ is a linear function of *x* and *x*^2^, with the highest degree being quadratic and no constant term. Therefore, according to the assumption of the logistic model, in the impact functions *f*_1_(*x*_*i*1_, *x*_*i*2_) and *f*_2_(*x*_*i*1_, *x*_*i*2_), a single variable has a maximum degree of two. Hence, since *f*_1_(*x*_*i*1_, *x*_*i*2_) describes the impact of *x*_*i*2_ on *x*_*i*1_, *f*_1_ must include *x*_*i*2_. Therefore, *f*_1_ can contain up to six terms: *x*_*i*2_, *x*_*i*2_*x*_*i*1_, $x_{i2}x_{i1}^{2}$, $x_{i2}^{2}$, $x_{i2}^{2}x_{i1}$, and $x_{i2}^{2}x_{i1}^{2}$, making a total of 63 possible combinations, calculated as $C_{6}^{1}+C_{6}^{2}+C_{6}^{3}+C_{6}^{4}+C_{6}^{5}+C_{6}^{6}=63$. Similarly, *f*_2_(*x*_*i*1_, *x*_*i*2_) can also be seen as a linear function containing six combinations of terms: *x*_*i*1_,*x*_*i*1_*x*_*i*2_,$x_{i1}x_{i2}^{2}$,$x_{i1}^{2}$,$x_{i1}^{2}x_{i2}$, and $x_{i1}^{2}x_{i2}^{2}$, resulting in 63 possibilities as well.

#### 4.1.2 Pattern detection process

Since the *f*_1_(*x*_*i*1_, *x*_*i*2_) and *f*_2_(*x*_*i*1_, *x*_*i*2_) of the three sets of equations may have different structures, it is necessary to test all possible structures and identify the common equations that can hold throughout the entire period. The testing steps are referenced in Section 3.2, and the specific process is as follows:

(1) Constructing difference equations. Transform the evolution models of the SI and CC into corresponding difference equations:


(5) $$\{\begin{array}{c} \Delta x_{i1}\left(n\right)=r_{i1}x_{i1}\left(1-\frac{x_{i1}}{K_{i1}}\right)+f_{1}\left(x_{i1},x_{i2}\right)\\ \Delta x_{i2}\left(n\right)=r_{i2}x_{i2}\left(1-\frac{x_{i2}}{K_{i2}}\right)+f_{2}\left(x_{i1},x_{i2}\right) \end{array}$$

This forms two sets of difference equations *S*_1_ and *S*_2_ containing the impact functions *f*, each consisting of 63 equations. Where Δ*x*_*i*1_(*n*) = *x*_*i*1_(*n*)−*x*_*i*1_(*n*−1) and Δ*x*_*i*2_(*n*) = *x*_*i*2_(*n*)−*x*_*i*2_(*n*−1), Δ*x*_*i*1_(*n*) and Δ*x*_*i*2_(*n*) are single-day data corresponding to the SI and CC, *n* = 1, 2, 3.

(2) Regression analysis. For each equation in set *S*_1_ and *S*_2_, it is necessary to conduct static simulation of global cyclic data first. From the structures qualified for the global cycle, we select the equation with the highest commonality (as many segmented data points as possible satisfy this equation) and then perform dynamic analysis to ensure that the selected structure is the optimal common structure.

(3) Regression test. After regression analysis of each equation, it is necessary to conduct regression tests. The criteria for the test are the same as in Section 3.2, including structural, correlation, and significance aspects. If the standards of the three aspects are met, the test is considered to pass.

(4) Selection of common structures. To select the impact function structures with the maximum commonality, we use the conformity rate (CR) introduced in Section 3.2 to record the three tests passed by dynamic regression analysis, and the proportion of equations whose structure coefficients are consistent with the sign of the structure. The higher the conformity rate, the greater the commonality of the impact function.

### 4.2 Regression analysis

After establishing the basic model and conducting preliminary structural detection, this study moved on to perform global static analysis. The objective of this stage was to conduct comprehensive static regression analysis on a total of 63 × 2 equations extracted from each data segment. Specifically, we conducted 63 × 2 regression analyses for the 3 sets of data, totaling 378 regression analyses, aiming to thoroughly evaluate each model.

During this stage, we subjected the results of each regression analysis to the three core tests mentioned in Section 3.2: structural test, correlation test, and significance test, ensuring the rigor of the analysis, and the accuracy of the results. This step is crucial for confirming the adaptability and universality of the selected model, as it involves verifying the applicability of the model under different time periods and conditions. Ultimately, among the 378 calculations, the two equations in the first segment had 15 and 26 structures passing, respectively, the second segment had 12 and 13 passing, and the third segment had 37 and 31 structures passing, respectively.

Next, we need to perform a preliminary screening of the results. Since the focus of this experiment is on studying the mutual influence between *x*_1_ and *x*_2_, we need to identify structures that can extract the factors of *x*_1_ or *x*_2_ and separate *x*_1_ and *x*_2_ from the structure. For example, for the impact function $f_{1}\left(x_{i1},x_{i2}\right)=-x_{i2}+x_{i1}^{2}x_{i2}$ = ($-1+x_{i1}^{2}$)*x*_*i*2_, extracting *x*_*i*2_ from the structure allows $-1+x_{i1}^{2}$ to be viewed as the specific impact of *x*_2_ on *x*_1_, facilitating a better understanding of the impact pattern.

Based on the above conditions, we preliminarily selected structures that meet the criteria and can be applied across all three phases, as shown in Table 3. Here, *a*_*n*_ > 0 and *b*_*n*_ > 0 for *n* = 1,2,3,4. From the table, it can be observed that in the impact function *f*_1_(*x*_*i*1_, *x*_*i*2_), all three segments contain the structure ($+x_{i1}^{2}x_{i2}$), and after extracting *x*_*i*2_, it becomes $x_{i1}^{2}$ plus a constant (where the constant is 0 when *i* = 1 and *i* = 3); in the impact function *f*_2_(*x*_*i*1_, *x*_*i*2_), all three segments contain the structure ($+x_{i2}^{2}x_{i1}$), and after extracting *x*_*i*1_, it becomes $x_{i2}^{2}$ plus a constant (where the constant is 0 when *i* = 2 and *i* = 3).

**Table 3:** Global static common structure.

**Impact function**	**First segment**	**Second segment**	**Third segment**
*f*_1_(*x*_*i*1_, *x*_*i*2_)	$+a_{1}x_{11}^{2}x_{12}$	-$a_{2}x_{22}+a_{3}x_{21}^{2}x_{22}$	$+a_{4}x_{31}^{2}x_{32}$
*f*_2_(*x*_*i*1_, *x*_*i*2_)	-$b_{1}x_{11}+b_{2}x_{12}^{2}x_{11}$	$+b_{3}x_{22}^{2}x_{21}$	$+{b_{4}x}_{32}^{2}x_{31}$

Through these comprehensive analyses, we have successfully identified structures with the highest commonality, which exhibit stable and consistent characteristics across different data segments.

### 4.3 Regression verification

After identifying common structures, we need to conduct dynamic regression analysis on these structures to verify their regularity and fit with the data across different segments. Specifically, we employed an incremental data dynamic analysis method: We used 70%, 80%, and 90% of the data in each segment as the base data, divided into three groups, and conducted dynamic regression analysis on the remaining 30%, 20%, and 10% of the data in each group, respectively. This approach allows us to observe the performance of the model at different data coverage rates to ensure the stability and reliability of the selected structures across the entire data range.

The dynamic regression analysis for each equation structure strictly adhered to the three core test criteria set earlier: structural, correlation, and significance tests, quantifying the fitting performance of each structure in different regression analyses using the conformity rate (CR). The results of dynamic regression analysis are presented in Tables 4–6.

**Table 4:** Regression analysis results with 70% basic data.

**Segment**	**Structure**	**Conformity rate (CR)**
First segment	$+a_{1}x_{11}^{2}x_{12}$	100%
	-$b_{1}x_{11}+b_{2}x_{12}^{2}x_{11}$	16.1%
Second segment	-$a_{2}x_{22}+a_{3}x_{21}^{2}x_{22}$	49.1%
	$+b_{3}x_{22}^{2}x_{21}$	55.5%
Third segment	$+a_{4}x_{31}^{2}x_{32}$	87.0%
	$+{b_{4}x}_{32}^{2}x_{31}$	100%

**Table 5:** Regression analysis results with 80% basic data.

**Segment**	**Structure**	**Conformity rate (CR)**
First segment	$+a_{1}x_{11}^{2}x_{12}$	100%
	-$b_{1}x_{11}+b_{2}x_{12}^{2}x_{11}$	24.2%
Second segment	-$a_{2}x_{22}+a_{3}x_{21}^{2}x_{22}$	73.0%
	$+b_{3}x_{22}^{2}x_{21}$	62.2%
Third segment	$+a_{4}x_{31}^{2}x_{32}$	100%
	$+{b_{4}x}_{32}^{2}x_{31}$	100%

**Table 6:** Regression analysis results with 90% basic data.

**Segment**	**Structure**	**Conformity rate (CR)**
First segment	$+a_{1}x_{11}^{2}x_{12}$	100%
	-$b_{1}x_{11}+b_{2}x_{12}^{2}x_{11}$	46.9%
Second segment	-$a_{2}x_{22}+a_{3}x_{21}^{2}x_{22}$	100%
	$+b_{3}x_{22}^{2}x_{21}$	70.3%
Third segment	$+a_{4}x_{31}^{2}x_{32}$	100%
	$+{b_{4}x}_{32}^{2}x_{31}$	100%

Upon further analysis of the dynamic regression results, it can be observed that except for the second impact function in the first segment, all other structures exhibited good fitting performance in the early stages of dynamic regression analysis (i.e., when the data coverage reached 70%). This early fitting excellence not only validates the effectiveness of the model but also demonstrates that these structures' CR values steadily increase in subsequent data analyses. Even for the second impact function in the first segment, the fitting rate reaches 46.9% with 90% data coverage, indicating that the impact functions with similar structures across the three groups meet the experimental requirements.

These findings provide crucial insights into understanding the structure of impact functions in dynamic environments, revealing the robustness of model structures in most scenarios. Therefore, based on the results of dynamic regression analysis, we finally determined the ultimate structure of the model. The complete model is as follows:


(6) $$\{\begin{array}{c} \frac{dx_{i1}}{dt}=r_{i1}x_{i1}\left(1-\frac{x_{i1}}{K_{i1}}\right)+a_{i}\left(x_{i1}^{2}+\varepsilon_{i}\right)x_{i2}\\ \frac{dx_{i2}}{dt}=r_{i2}x_{i2}\left(1-\frac{x_{i2}}{K_{i2}}\right)+b_{i}\left(x_{i2}^{2}+\delta_{i}\right)x_{i1} \end{array}$$

Where *a*_*i*_>0, *b*_*i*_>0, ε_*i*_ and δ_*i*_ can be 0, for i = 1,2,3. By introducing ε_*i*_ and δ_*i*_ as adjustment terms, we further enhance the flexibility of the model, enabling it to more accurately simulate the complex dynamic changes in the real world. This refined model is not only more mathematically precise but also more interpretable and predictive in practical applications.

## 5 Discussion

### 5.1 Model mechanism explanation

This study constructed a model based on large-scale behavioral data to analyze the non-linear correlation between COVID-19 search behavior and confirmed cases. Unlike traditional mathematical models, the patterns of this model are not easily discernible merely by observing the equation structures. To explore its underlying principles, we need to conduct an in-depth analysis of the model.

We transform the common equations as follows:


(7) $$\{\begin{array}{c} \frac{dx_{i1}}{dt}=r_{i1}x_{i1}\left(1-\frac{x_{i1}}{K_{i1}}\right)+\theta_{1}x_{i2}\\ \frac{dx_{i2}}{dt}=r_{i2}x_{i2}\left(1-\frac{x_{i2}}{K_{i2}}\right)+\theta_{2}x_{i1} \end{array}\;$$

**Figure 5:**
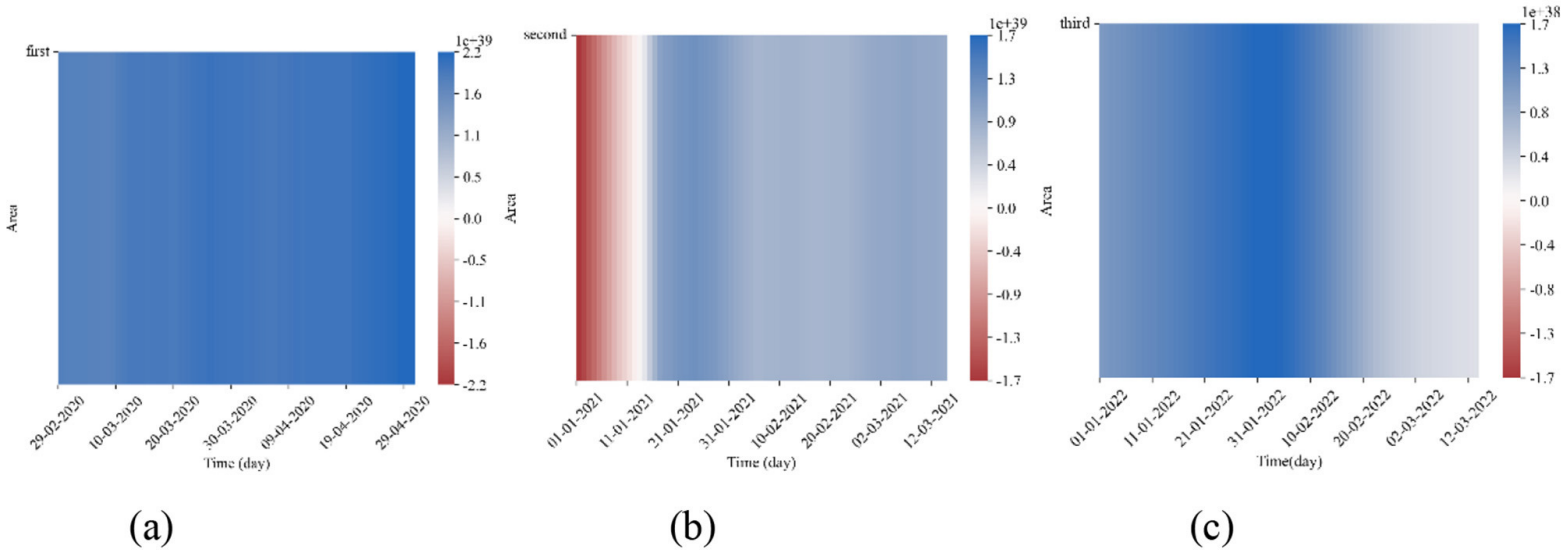
Heatmap of θ_1_(*t*) Magnitudes. **(a)** Segment 1. **(b)** Segment 2. **(c)** Segment 3.

**Figure 6:**
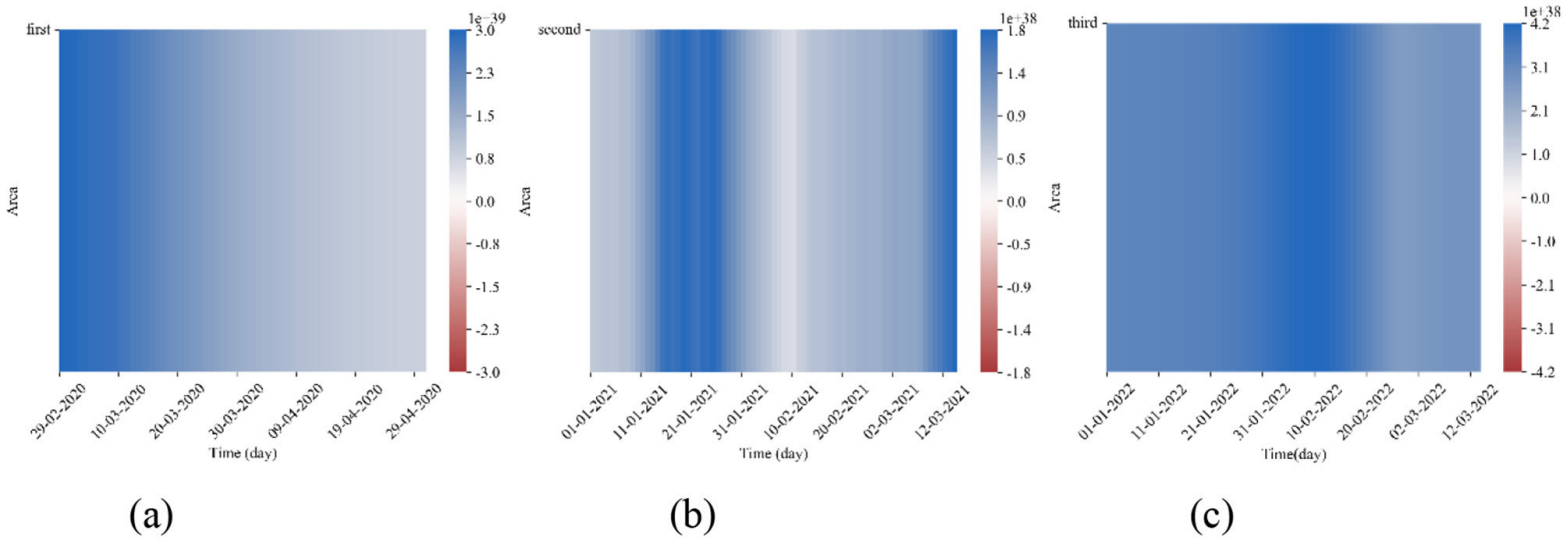
Heatmap of θ_2_(*t*) Magnitudes. **(a)** Segment 1. **(b)** Segment 2. **(c)** Segment 3.

Where $\theta_{1}\left(t\right)=\;a_{i}\left(x_{i1}^{2}\left(t\right)+\varepsilon_{i}\right)\;$ and $\theta_{2}\left(t\right)=\;b_{i}(x_{i1}^{2}(t)+\delta_{i})$. θ_1_(*t*) represents the impact of CC on SI at time *t*, while θ_2_(*t*) represents the impact of SI on CC at time *t*. Next, we start from 80% of the data volume of the three segments to calculate the effects of θ_1_(*t*) and θ_2_(*t*), and draw a heatmap. The results are shown in Figures 5, 6. Notably, the color bar units for Figures 5a, b, 6a are 1e39, while the units for the remaining figures are 1e38.

Observing the heatmaps in Figures 5, 6, except for the early section of the heatmap for θ_1_(*t*) in the second segment, which appears red, the majority of the heatmap is blue. This indicates that the effects of θ_1_(*t*) and θ_2_(*t*) are generally promoting. This finding is similar to the study by Galido et al. (14). We can preliminarily conclude that there exists a mutually reinforcing relationship between the SI and CC. Specifically, the growth in SI to some extent drives the increase in CC, while the rise in confirmed cases also stimulates people's demand for searching information about the epidemic.

### 5.2 Mechanism of SI and CC

At this point, the study has successfully identified and quantified the mutual influence mechanism between the rate of change of the SI and CC. Through the established non-linear model, we have revealed the mutually reinforcing effects of these two factors in the context of epidemic spread. Next, we will further explore the specific manifestations and underlying mechanisms of this relationship.

As the pandemic evolves and the number of confirmed cases rises, the uncertainty and danger of the epidemic compel people to generate a large demand for information related to the epidemic. This demand includes not only the development status of the epidemic and prevention measures but also treatment methods and social impacts (19). From a psychological perspective, this behavior is actually a psychological defense mechanism that people use when facing uncertain things and potential threats (20). Information search can provide a certain degree of psychological comfort (21). In addition, as the number of confirmed cases continues to rise, media coverage of the epidemic becomes more intensive, further promoting information search behavior (22). Media reports not only provide the latest developments in the epidemic but also reflect the government's and health departments' responses to the epidemic and measures taken. These pieces of information are crucial for the public to understand the epidemic situation and take corresponding measures. This provides a wealth of data basis for people's search behavior, exacerbating their search for information.

In addition, our team member Wang et al., through empirical analysis of online medical consultations and search data, demonstrated that this model exhibits strong robustness both during the pandemic and in normalized periods (23). This research suggests that while the model was validated in different application scenarios, the similar non-linear structure effectively adapts to dynamic changes across various stages, providing strong support for further research on public health events.

Future studies can build upon the experimental framework of this research to expand its application to other contexts. The focus of this study is on establishing a stable self-impact mechanism that minimizes the influence of external factors, thereby revealing deeper interactions between variables. As the model's structure and operators remain unchanged across different scenarios, even when the data dimensions increase, the addition of corresponding equations allows for continued effective analysis. This approach not only accommodates the complexity of multidimensional data but also lays a solid foundation for applying this research to a broader range of public health events in the future.

However, in some cases, excessive information search may lead to information overload, increasing people's anxiety and panic. This panic may prompt some people to overreact, such as hoarding supplies and panic buying medication, which may inadvertently exacerbate social instability (24).

With increasing attention to the epidemic, the increased search behavior not only enhances people's understanding of the epidemic but also promotes their understanding and adoption of epidemic prevention and control measures. For example, information obtained through searches helps people understand the importance of properly wearing masks, hand hygiene, and maintaining social distance, among other preventive measures. These behaviors to a certain extent help control the further spread of the epidemic. This awareness of epidemic prevention prompts people to actively seek medical assistance and nucleic acid testing when related symptoms appear early, as more potential cases are confirmed, leading to an increase in confirmed cases.

### 5.3 Insights

At this point, this study has successfully identified and quantified the mutual influence mechanism between the rate of change in SI and CC, providing a search data-based predictive tool that can assist public health authorities in monitoring the development of the epidemic and taking appropriate response measures in a timely manner. Compared to traditional epidemic monitoring methods, search data offers higher timeliness and flexibility, providing rapid feedback to health departments, especially in the early stages of an epidemic or when there are delays in reporting confirmed cases. It enables earlier detection of potential changes in the epidemic. In this process, the flow of information acts as a bridge linking public reactions and epidemic development, serving as both a mechanism for the social system to respond to the epidemic and a key factor influencing the trajectory of the epidemic (25).

In practical applications, search data is a valuable resource for public health monitoring. As the epidemic develops, the public's demand for related information changes in phases, and the volume of these searches can reflect the public's level of concern about the epidemic. For example, if a region shows a spike in searches for keywords related to symptoms, it may indicate a potential outbreak risk in that area (26). The non-linear model established in this study, based on the lifecycle theory, divides the epidemic progression into three phases: the “latent period,” the “outbreak period,” and the “decline period,” which can be achieved by calculating the higher-order derivatives of the model. The core value of the model lies in its ability to accurately capture the dynamic relationship between SI and CC during each phase, thereby providing customized strategic recommendations for public health authorities.

(1) **Latent period**: the characteristic of the latent period is that the number of confirmed cases is relatively low, but public attention to relevant information begins to rise. During this phase, the search volume typically fluctuates less. Through model analysis, health departments can monitor search trends for specific keywords, identify or predict potential signals of an epidemic outbreak, and formulate early response strategies, such as enhancing the promotion of protective measures or disseminating more prevention knowledge through public health platforms. By addressing the public's need for epidemic information early on, the growth of epidemic-related search volume can be indirectly reduced, prompting people to take preventive measures during the incubation period and thus lowering the occurrence of potential confirmed cases.
(2) **Outbreak period**: when the epidemic reaches the outbreak phase, search volumes typically spike, and public attention to the epidemic peaks. In this phase, the model can help predict the further development of the epidemic by analyzing the relationship between search data and confirmed case numbers. For example, if there is a sudden surge in searches related to “COVID-19 symptoms” in a particular region, it may indicate that the region has entered a high-risk outbreak period, and confirmed cases may sharply rise afterward. Health departments can use the model's predictions to take proactive, strong preventive measures, such as enhancing isolation protocols, closing high-risk areas, and intensifying health inspections. By doing so, health departments can more flexibly and timely adjust their emergency response strategies, effectively curbing the spread of the epidemic and minimizing the impact of the outbreak phase.
(3) **Decline period**: once the epidemic is brought under control and enters the decline phase, the number of confirmed cases begins to decrease, and public attention to the epidemic also diminishes, leading to a gradual drop in search volumes. At this stage, the model can assist health departments in identifying the trend of epidemic decline and guide subsequent policy adjustments. Although the risk of epidemic transmission is lower during this phase, to prevent a resurgence, health departments still need to monitor search data to gauge the continued public attention to epidemic control measures. If the search volume begins to rise again, it may indicate a decrease in vigilance in certain regions or among specific groups. Health departments can use this signal to promptly strengthen health education and public health campaigns to maintain public awareness of epidemic prevention.

By combining daily search volume with the changes in confirmed case numbers, public health departments can monitor the evolution of the epidemic in real-time, accurately identifying the characteristics of each phase and implementing targeted interventions accordingly. This non-linear model-based forecasting method not only provides a more precise time window to help health departments detect potential risks of the epidemic in advance but also allows for dynamic adjustments in prevention and control strategies, making emergency responses more proactive and targeted. Changes in search data provide public health departments with faster and more sensitive feedback compared to traditional monitoring methods, helping to avoid delays in decision-making relative to the epidemic's progression, thereby minimizing the spread of the public health crisis and its social impact.

However, analyzing data using internet search data also poses challenges. Nowadays, search engines are becoming increasingly intelligent and provide personalized search results based on users' search history and location, among other factors (27). This leads many people to fall into information bubbles. Once search engines identify users' preferences and interests, users receive tailored recommendations that greatly affect the accuracy and objectivity of search data. To avoid this situation, we need to improve the quality of information supply in searches. This includes providing scientifically accurate and high-quality search information, as well as intelligently recommending more popular science and rumor-refuting information, preemptively compressing the spread space of false and provocative information, and avoiding mass panic and social disorder (28). These measures cannot only stabilize public emotions, reduce unnecessary panic and anxiety but also promote the public's rational understanding and response to the epidemic. At the same time, the public can also use structured information more frequently to enhance search quality and thereby achieve better information gathering results (29).

Furthermore, non-linear detection based on differential equations provides us with a clearer and more precise analytical tool. Compared to traditional data mining or machine learning models, differential equation models have advantages in quantifying the degree of influence and explaining the mechanism of influence (30). By establishing non-linear models, we can more accurately capture the interaction between different factors, thereby more accurately predicting the development trend and risk factors of the epidemic. Such models cannot only help policymakers make data-based decisions more quickly but also enhance the public's understanding and comprehension of the epidemic, thus more effectively responding to public health crises.

In conclusion, by establishing interaction models and combining non-linear detection based on differential equations, we cannot only achieve predictions of epidemic trends but also provide scientific basis for health departments and decision-makers, thereby more effectively coping with possible future public health challenges.

## 6 Conclusion

This study conducts an in-depth analysis of the relationship between internet search behavior and confirmed COVID-19 cases, proposing a rate-of-change-based non-linear model. The model demonstrates that search behavior can reflect the public's attention and information needs during sudden events. Unlike previous models that primarily focused on simple associations between variables, this study innovatively quantifies the dynamic interaction between search behavior and confirmed cases through rate-of-change analysis, uncovering their mutual influence mechanisms.

The research constructs a mathematical framework for epidemic transmission based on the Logistic model, utilizing cumulative data from the epidemic search index and confirmed cases. By introducing the concept of “evolution,” it identifies the equation structure that best explains the interaction between search indices and confirmed cases. The core innovation lies in not only examining the non-linear relationship between variables but also capturing the dynamic feedback mechanism between search behavior and confirmed cases through their rates of change, offering new insights into understanding the complex mechanisms of epidemic transmission.

Through model fitting and analysis, the study finds that an increase in search indices can drive up confirmed cases, and vice versa. This cross-influence validates the effectiveness of the rate-of-change model in revealing complex relationships. These findings provide crucial theoretical support for optimizing information dissemination and public health interventions. For instance, guiding public search behavior can more effectively control epidemic transmission trends.

However, this study has certain limitations. First, although Baidu, as China's primary search engine, has wide coverage and represents most search behaviors, internet search data is influenced by personalized recommendation algorithms and the filter bubble effect, which may introduce bias and affect the generalizability of the results. Future research should explore ways to mitigate the influence of personalized algorithms on data analysis. One possible approach is to segment search data based on user characteristics and assign differentiated weights to different user groups. Specifically, for professional users who frequently focus on the field of infectious diseases, the search data generated by them should be given lower weight, while for ordinary users who are less exposed to this field, higher weight should be assigned. This approach can enhance the objectivity of the study results and improve the accuracy of the analysis.

Secondly, in this study, we used the number of confirmed cases as the primary measure of epidemic transmission. However, the reporting of confirmed cases is often influenced by timeliness and reporting delays, especially during epidemic peaks. The delay in reporting cases may result in a gap between actual case numbers and reported case numbers, which directly affects the relationship between search data and confirmed data, as well as the prediction accuracy of the model. Future research could consider integrating multi-source data, including case reports from authoritative hospital centers, to improve the accuracy of confirmed case data.

Finally, since this study focuses on exploring the regularities in the rates of change between variables and their impact mechanisms, the depth and breadth of the data are somewhat limited. In terms of data depth, the study primarily focuses on the temporal dimension and does not fully account for geographic differences in epidemic transmission, as well as the impact of emotional social reactions (such as anxiety and uncertainty) on information search behavior. Future research should further explore how to incorporate regional differences and emotional responses into the study to gain a more comprehensive understanding of the influence mechanisms between information search behavior and epidemic transmission. In terms of data breadth, this study mainly relies on Baidu Index and data from the National Health Commission of China, which are highly representative within China. However, given the differences in epidemic monitoring and data reporting practices across countries, future research should consider referencing similar data sources from other countries, particularly those with authoritative and complete time-series data sources. If other countries also have similar high-quality data (such as Google search and other public health monitoring data) with a sufficient time span, researchers could adopt the methodology used in this study and expand it to other regions for comparative analysis, thereby validating the generalizability and cross-country applicability of the model.

## Data Availability

The original contributions presented in the study are included in the article/Supplementary material, further inquiries can be directed to the corresponding author.
