# Supplementary figures and images for: Non-linear correlation analysis between internet searches and epidemic trends
Source: Front Public Health. 2025 Apr 4;13:1435513. doi: 10.3389/fpubh.2025.1435513 (PMC12006183; doi:10.3389/fpubh.2025.1435513)

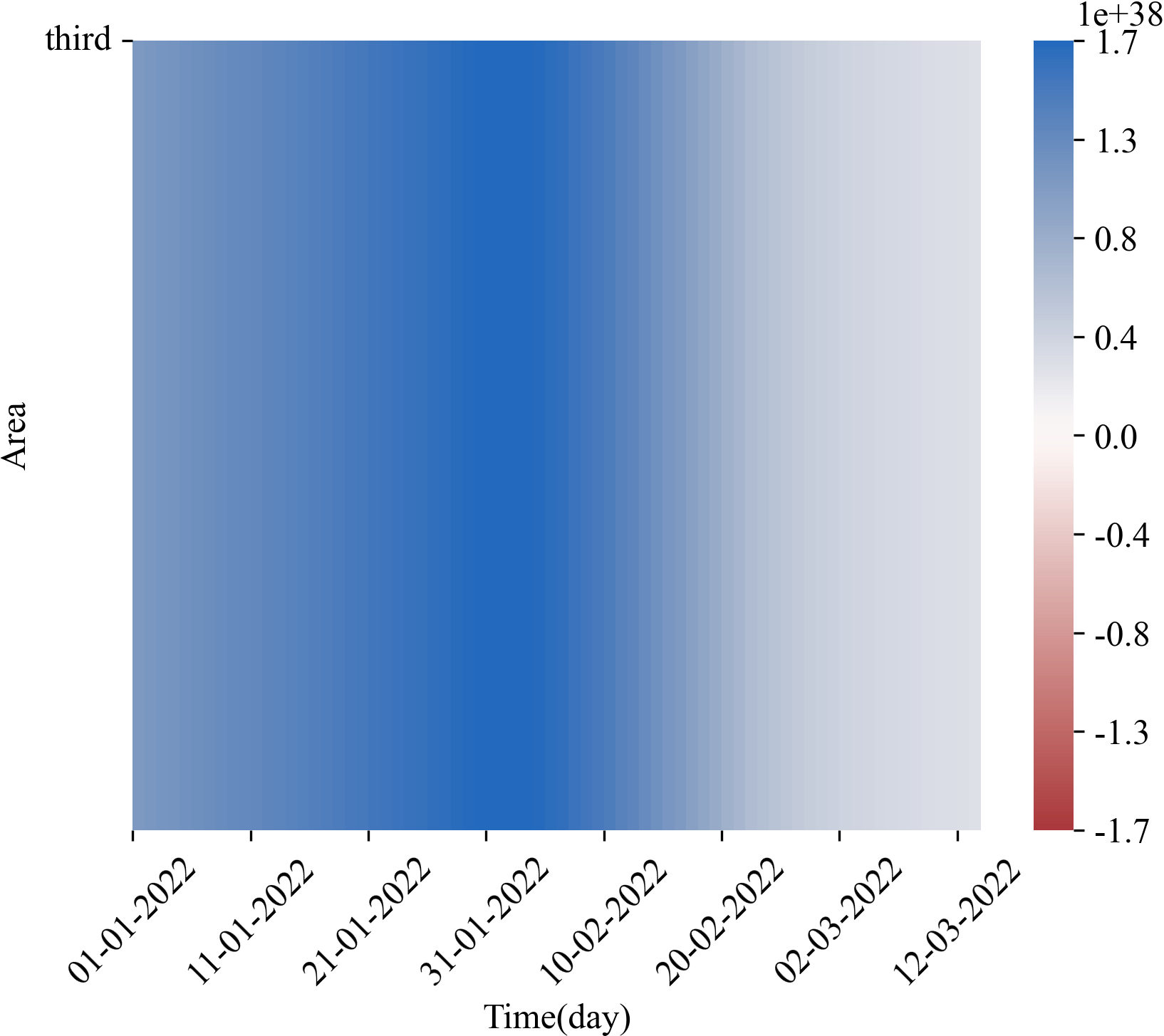

Supplement: Supplementary file 2 [file Image_1.tiff]
